# How does feedback in mini-CEX affect students’ learning response?

**DOI:** 10.5116/ijme.580b.363d

**Published:** 2016-12-19

**Authors:** Sulistiawati Sudarso, Gandes Retno Rahayu, Yoyo Suhoyo

**Affiliations:** 1 Department of Medical Education, Faculty of Medicine, Mulawarman University, Indonesia; 2 Department of Medical Education, Faculty of Medicine, Universitas Gadjah Mada, Indonesia

**Keywords:** Feedback, mini-CEX, learning response

## Abstract

**Objective:**

This study was aimed to explore students’ learning response toward feedback during mini-CEX encounter.

**Methods:**

This study used a phenomenological approach to identify the students’ experiences toward feedback during mini-CEX encounter. Data was collected using Focus Group Discussion (FGD) for all students who were in their final week of clerkship in the internal medicine rotation. There were 4 FGD groups (6 students for each group). All FGD were audio-taped and transcribed verbatim. The FGD transcripts were analyzed thematically and managed using Atlas-ti (version 7.0).

**Results:**

Feedback content and the way of providing feedback on mini-CEX stimulated students’ internal process, including self-reflection, emotional response, and motivation. These internal processes encouraged the students to take action or do a follow-up on the feedback to improve their learning process. In addition, there was also an external factor, namely consequences, which also influenced the students’ reaction to the follow-up on feedback. In the end, this action caused several learning effects that resulted in the students’ increased self-efficacy, attitude, knowledge and clinical skill.

**Conclusions:**

Feedback content and the way of providing feedback on mini-CEX stimulates the students’ internal processes to do a follow-up on feedback. However, another external factor also affects the students’ decision on the follow-up actions. The follow-ups result in various learning effects on the students. Feedback given along with summative assessment enhances learning effects on students, as well. It is suggested that supervisors of clinical education are prepared to comprehend every factor influencing feedback on mini CEX to improve the students’ learning response.

## Introduction

Feedback is the foundation of effective clinical teaching. It is particularly significant to reinforce good performance, correct the poor performance, as well as to identify the area that needs to be improved.^1^^-^^3^ It is evident that feedback has a positive influence on the improvement of a students’ performance.^4^

Each feedback may be treated differently by students. It may accurately correct their errors, but it may also still lead to no change on their next or future tasks.^5^ Taylor and Hamdy^6^ describe a theory about adult learning where feedback is a crucial phase.  Feedback given by a teacher will reinforce the students’ existing knowledge or oblige the learner to reconsider it in the light of new information.

Mini-CEX (mini clinical evaluation exercise) is one of the assessment methods that provide feedback immediately afterwards. In mini-CEX, the clinical supervisor can provide feedback to the students based on direct observation. Students have the opportunity to be assessed in various clinical settings and receive feedback by multiple assessors.^7^^-^^10^ Existing research shows that mini-CEX has an influence on the learning process. The use of mini-CEX can enhance the students' learning experience. Because of its recurrent nature, mini-CEX has increased the students’ study time and motivation to learn.^11^ Students find that mini-CEX is useful as a learning tool. The mini-CEX allows for real-time, objective, formative feedback. This assessment process provides feedback to the students with a goal of improving their clinical capabilities, which may positively reinforce their experiences. Once they finish the mini-CEX and receive the feedback, they can identify their weak areas. It reinforces and gives students an opportunity to go back and look at any particular area regularly.^12^ Weller and colleagues^13^ investigated the mini-CEX method and found that students appreciated being assessed against the expected standard for their level of training, and liked to know how they were compared with their peers.

Mini-CEX has a positive effect on students’ learning process.^14^ Research done by de Lima and colleagues^14^ was designed to illustrate how residents perceive the mini-CEX as an assessment tool and its influence on their approach to learning and studying. The result showed that with mini-CEX, students used a deep learning approach.  It is also supported by Al-Kadri and colleagues^15^ research about the effect of workplace-base assessment (mini-CEX, DOPS, and CBD) on the medical students’ learning approach. The result stated that feedback in workplace-base assessment helped students to identify their weaknesses and the areas where they really shine, as well as give them ideas on ways to improve.

Several studies have been published and have proven that students have a positive experience both in terms of knowledge and skills when mini-CEX is implemented. However, the mechanism of how feedback, as an integral component in mini-CEX, affects the learning process is still left unanswered. Therefore, this study was conducted to explore the students’ learning response toward feedback during mini-CEX encounter.

## Methods

### Study design

Phenomenology refers to the description of one or more individuals’ consciousness and experience of a phenomenon. In this study, the phenomenological study was utilized to identify the students’ experiences toward feedback during mini-CEX encounter.

Study participants, sample size, and sampling methods

This study consisted of 24 participants (9 males and 15 females). Participants were divided into 4 groups (6 people per group). Participation was voluntary and informed consent was obtained beforehand. Students undergoing clerkship in the internal medicine rotation were invited to participate in the study.   Students repeating clerkship in the internal medicine rotation at the time of the study were excluded. The study was approved by the Medical and Health Research Ethics Committee (MHREC) of Faculty of Medicine, Gadjah Mada University Dr. Sardjito General Hospital.

### Data collection

Data was collected using Focus Group Discussion (FGD). There were 4 FGD groups (6 students for each group). The FGD was conducted twice for each group. The first FGD was conducted on the last clerkship week of the internal medicine rotation in order to prevent recall bias. The second focus group meeting was held as a means to do member-checking and to clarify the issues that had been delivered in the first FGD, or not presented in the first meeting. All FGDs were conducted by the first investigator accompanied by a note-taker. The focus group discussions were audio-taped and then transcribed by the first investigator. The transcription was then checked by another independent person in order to make sure that it was similar to the recording. This process was performed on all FGDs.

### Procedure

Mini-CEX has been used in the Faculty of Medicine, Mulawarman University since 2011. The supervisors have been trained to conduct mini-CEX along with other assessment methods in clinical education. The faculty also briefed the students about mini-CEX before entering the clerkship.

This study was performed in an internal medicine rotation. The duration of the internal medicine rotation was 12 weeks. This rotation has 7 divisions, with each division implementing the mini-CEX method.  Mini-CEX contributed 10% of the students’ final grades. There were only 3 divisions that met the standard criteria of mini-CEX, namely the pulmonology division, the endocrine division, and the cardiology division. Probing technique was used during the FGD. Questions asked during the FGD were listed below:

1.     Did you receive feedback during mini-CEX? (If not, what did you do? Why? If yes, what kind of feedback did you receive?)

2.     How was your acceptance of the feedback given by the supervisor during the sessions of the Mini-CEX?

3.     What learning processes did you apply after receiving feedback on the Mini-CEX?

### Data analysis

The FGD transcripts were analyzed thematically and managed using Atlas-ti (version 7.0) computer software. Data was analyzed by determining the open coding first, according to statements found in the transcript. Data from open coding were grouped into categories that would eventually be regrouped into theoretical codes. Theoretical codes that had been formulated were then regrouped into major categories.

The coding process was performed by the first investigator and one colleague with a background in medical education. The two coders performed the process independently. After that, they met to make an agreement on the emerging themes. If there was a discrepancy between the two coders, it was discussed between the coders to reach an agreement. The results of the coding process were then reviewed by GRR and YS.

## Results

Three major themes arose from the focus groups, i.e. feedback on the mini-CEX, students’ response to the feedback during mini-CEX, and its effect on learning. The following description of the themes was the summary of what the students expressed, as illustrated by selected quotations and translated as closely as possible from Bahasa Indonesia to English.

### Feedback during mini-CEX

Feedback during mini-CEX has two important components: the content and the way of providing feedback.

#### The content of feedback

Supervisors informed the students about their achievements of competence in history taking, physical examination, diagnosis, differential diagnosis, supporting investigation, management and patient education.

“. . . from the chief complaints, then a history of the present illness, past medical history, psychosocial history, then up to medications . . .” (No 2, female, focus group 1)

 “. . . supervisor was explaining the correct way to do the examination. Investigations should not be done carelessly; there must be a (theoretical) basis. Differential diagnosis must be related to the previous statement . . .” (No 3, female, focus group 2)

 “. . . supervisor taught us how to educate patients about their disease . . .” (No 4, female, focus group 3)

Feedback given by the supervisor also contained the performance gap that occurred when students examined patients during mini-CEX. The supervisor told the students about what they did well, what needed improvement and taught general principles.

“. . . If I have a fault, the doctor will correct it by telling me how to do it the right way. . .”  (No 2, female, focus group 3)

#### The ways of providing feedback

The supervisor provided feedback in two ways: directive and facilitative.

#### Directive feedback

Directive feedback includes giving an example, immediate correction, guiding the examination and giving explanations.

“. . . doctor is showing how to perform the correct examinations . . .” (No 6, female, focus group 1)

“. . . I misplaced the stethoscope on the chest of the patient, so the supervisor told me ‘the stethoscope should be placed here’ . . .” (No 1, female, focus group 1)

#### Facilitative feedback 

Facilitative feedback includes asking questions, discussion, giving reference, directing what needs to be learned. In addition, the supervisor also provided a task if the students could not answer questions during mini-CEX session, and gave the order to do a follow-up on the case they handled during mini-CEX.

 “. . . the supervisor would give questions. He will confirm whether our response has led to a diagnosis or not . . .” (No 2, female, focus group 3)

 “. . . supervisor asked me 'do you know about cardiac asthma?' because I did not know the answer, the supervisor give an assignment to me . . .” (No 2, female, focus group 4)

#### Students’ response to feedback during mini-CEX

Students’ response to feedback during mini-CEX in this study is defined as the students’ conditions after receiving feedback and their follow-up actions on the feedback. Feedback stimulates self-reflection, improves motivation, and emotional response. These responses triggered the students to do a follow-up on the feedback.

#### Stimulated self-reflection

Feedback in mini-CEX makes the students aware about what is right or wrong, realize their shortcomings, and makes them aware of the lack of preparation before taking the mini-CEX.

“. . . we know what is lacking and what is right . . .” (No 4, male, focus group 2)

“. . . with feedback, I became aware that my preparation is lacking, so in the future I will be more prepared . . .” (No 5, male, focus group 1)

#### Increased motivation

Students showed increased motivation as the mini-CFX method enhanced their curiosity, stimulated their passion, and encouraged students to learn.

“If there is feedback, our curiosity is ignited.” (No 1, female, focus group 2)

“If the supervisor gives an additional task, we will immediately do it. The task increases our passion (to study). It means that the supervisor responded to what we did incorrectly.” (No 2, female, focus group 3)

“Feedback makes me learn. If there is no feedback, I do not learn.” (No 5, female, focus group 4)

#### Emotional response

Students felt a positive emotional change when receiving feedback. Feedback made students feel happy, valued, and cared for by the supervisor.

“Even when I was blamed, I'm a happy doc. (F: why?) I feel that the doctor cares for us.” (No 4, male, focus group 4)

“. . . so if we have to do something, we feel more appreciated. There is appreciation and (we) feel happy just like that” (No 2, female, focus group 3)

#### Follow-up actions on the feedback

Not all students who received feedback did follow-up actions regarding the feedback. Students’ actions after receiving feedback varied depending on the content, for example: work on a task; practice the skills again following the supervisor’s advice; seek help by asking friends, another supervisor, or a general practitioner; search on the internet; or read a book. Feedback from the supervisor was listed as a priority by the students in their learning process.

“I immediately do the follow-up. I think that the doctor’s order is important, it makes me immediately find. . .” (No 5, female, focus group 3)

“If supervisors give an example, I can apply it to the patients (in the ward). I was taught about how to do crepitation examination on patients with osteoarthritis. When I met with OA patients, I immediately looked for a sign of crepitation” (No 4, male, focus group 4)

If the feedback has significant consequences to the student, they will definitely do a follow-up action as a response to the feedback. Supervisors would check whether the students do what had been told to them and determined their passing grade based on it. In addition to the consequences, motivation also plays a role in the follow-up actions. However, even if the feedback did not have consequences, students were still motivated to do follow-up actions, as they were driven by curiosity and desire to prove themselves to the supervisor. In conclusion, without consequences or motivation, students may not do any follow-up actions on the feedback.

“Supervisor will not let us pass, until we do the work they ask for. So, the task must be done.” (No 5, female, focus group 4)

“. . . supervisor did not check the task, so it was not done. . . There is also another writing task which has to be completed. If you cannot give the answer, the supervisor will give another question or task. . .” (No 5, male, focus group 1)

“I still do the task . . . I want to prove to the supervisor that I have completed the task” (No 5, female, focus group 4)

#### Learning effects

Learning effects are defined as the effects perceived by the students on their learning process after doing follow-up actions on the feedback in mini-CEX. In this study, the learning effects include the increase in self-efficacy, attitude, knowledge and clinical skills of the students.

#### Increased self-efficacy

Increased self-efficacy refers to the increase in confidence and readiness to face the same case.

“. . . we feel more confident, we know what we need to explore from a patient. . .” (No 6, female, focus group 6)

“. . .  so, if we examine the patient with the same symptoms as before, we will not go wrong again in the examination because of the feedback we got before. . .” (No 5, male, focus group 2)

#### Increased attitude

Increased attitude means that the students showed increased attention to the patients.

 “At the previous division, we are only concerned about the examination. We do not pay attention to whether the patient knows about the disease or not, whether the patient has been taking the medicines or not, we do not really care. But after a few times of mini-CEX, we become more attentive . . .” (No 4, female, focus group 3)

#### Increased knowledge

In this study, an increase in knowledge shows that there was an improvement in the students’ systematic mindsets and insights, keeping the material in mind, and understanding the theory.

“systematic thinking from anamnesis, examination until differential diagnosis.” (No 2, female, focus group 1)

“. . . the feedback is not just about physical examination but also the theory. We become more knowledgeable about the theory.”  (No 4, male, focus group 1)

#### Increased clinical skills

Another learning effect includes the increase in the students’ clinical skills. The students’ ability to do history taking and physical examination increased after implementing feedback from the supervisor.

“. . . supervisor gives input that the anamnesis should be like this, so that the next anamnesis will be improved” (No 2, female, focus group 1)

“. . . the examination is to be more systematic. In addition to finding lung irritation, we should also find other disorders, such as disorders of the liver. . .” (No 1, female, focus group 3)

### The model of students’ response mechanism to feedback on the mini-CEX

There are two important components of the feedback in mini-CEX, namely the content and the way of providing feedback.

Feedback content is useful to describe the students’ performance in the achievement of competence and performance gap, while the way of providing feedback can be divided into two categories, i.e. directive and facilitative.

**Figure 1:**
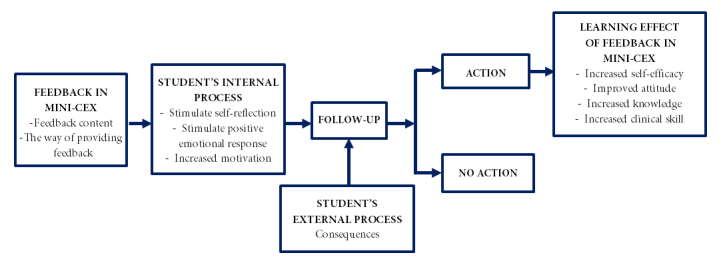
The model of student’s response to feedback in mini-CEX

Both feedback content and the way of providing feedback are imperative in encouraging students’ internal processes. The feedback given by a supervisor in the mini-CEX ultimately affects the students’ internal processes. The internal processes stimulated by feedback include self-reflection, motivation and positive emotional response. These internal processes result in how the students do a follow-up on the feedback (Figure 1).

This study also finds that in addition to internal processes, another external process, namely consequences, can also trigger the students to do a follow-up on mini-CEX feedback. Mini-CEX as a summative assessment gives certain consequences to students. If the feedback is not implemented, students will not receive grades and not be able to take their final exams. In the end, the students will need to repeat the rotation in the department.

## Discussion

Several previous studies indicate that the mini-CEX affects the learning process. Mini-CEX is a useful assessment tool and has a positive influence on the learning process.^12^^-^^14^ By doing mini-CEX repetitively, students will spend more time in practicing history taking and physical examinations, and also increase their learning time.^11^ Mini-CEX provides a positive experience, both in terms of knowledge and clinical skills.^16^

This study gives insight about the mechanism that happens during the students’ learning process after receiving feedback during mini-CEX. Feedback in mini-CEX can stimulate the students’ internal process, i.e. self-reflection, emotional response and motivation. These internal processes will encourage the students to take action based on the content of the feedback. The study also shows the role of external factors, namely consequences, that also affects the follow-up on the feedback. Follow-up on the feedback will increase self-efficacy, attitude, knowledge and clinical skills.

The results of this study support the previous studies, especially regarding feedback. This study finds that feedback in mini-CEX can stimulate the internal process of the students. This study reinforces the statement that the feedback can stimulate an individual’s internal process.^17^^, ^^18^

Feedback, especially in clinical education, can be effective and accepted by the students if the content is given immediately and individually after the performance by the people who observe the performance directly. The content should ideally be directed on the observed performance and how to improve it.^19^^-^^21^ In this study, students received feedback from the supervisors who observed them directly during mini-CEX. Feedback was provided individually and carried out immediately after the performance.

Students are most likely to accept feedback in which the content is directed toward the achievement of competence and performance gap, or in other words, provides information on the students’ performance. This reinforces the statement that feedback on performance is better received by the students rather than feedback about the "self".^19^^-^^21^ Feedback is significantly more effective when it provides details on how to improve the performance rather than just telling the students if the performance is good or not.^22^ In this study, the way of providing feedback, both in directive and facilitative ways, can help students correct their mistakes and thus improve their performance.

Feedback will stimulate students to do self-reflection.^6^ This also occurs in the feedback on mini-CEX.^12^^,^^23^

Feedback has also been shown to increase the students’ learning motivation.^18^^,^^23^ This study proves that feedback in mini-CEX can stimulate self-reflection and motivation.

Receiving feedback is a process that involves emotions.^19^^,^^24^^,^^25^ The results of this study add that the emotional responses that arise in the mini-CEX is a positive emotional response such as feelings of pleasure, valued, and cared for. This might be caused by the appreciation of students to the supervisor who provided feedback.  Suhoyo and colleagues^26^ stated that cultural aspects influence the feedback reception. Indonesia is a country with a high power distance. In countries with high power distance, students will follow the direction set by the teachers. Feedback given by the teacher will be appreciated by the students.

This study shows that external factors also influence the actions of the students toward feedback. In this study, the external factor is consequences. Mini-CEX can be either a formative assessment or summative assessment.^12^^,^^16^^,^^27^ Mini-CEX as a summative assessment will bear a consequence (appraisal of impact).^28^ This study noted that consequences affect whether a student will do a follow-up or not. In the end, these actions will lead to learning effects.

Research shows that the mini-CEX can increase self-efficacy,^16^ knowledge,^14^^,^^16^ and clinical skills.^12^^,^^16^ This study proves that feedback in mini-CEX is the one part that encourages learning, resulting in a learning effect that will increase self-efficacy, attitude, knowledge and clinical skills.

This study provides additional results and support to the research conducted by Ciliers and colleagues^29^ about the impact of summative assessment on the students learning. According to Ciliers and colleagues^29^ task demand and system design will affect the students learning. This study shows that in addition to task demand and system design, feedback also plays a role in the students’ learning process. Moreover, feedback on summative assessment can also affect the students’ learning process.

This study also adds to the mechanisms of learning effects on summative assessment. Ciliers and colleagues^29^ stated that the learning effects may be influenced by impact appraisal (consequences), response appraisal, perceive agency, and interpersonal factors. This study adds that in addition to these four factors, the mechanism of learning effects is also influenced by self-reflection and a positive emotional response.

The limitation of this study is that it only used FGD to obtain data on students’ learning responses. Therefore, no other data was available to confirm the learning responses obtained from FGD. It is recommended that further research be conducted using other methods to examine the response of learning, for example by using a log-book to criticize learning response after receiving the feedback, followed by in-depth interviews. As this research was conducted in Indonesia (a country with high power distance), other researchers may conduct research in other places with similar characteristics to see whether similar results generated by this study is present, as well.

## Conclusions

Feedback in mini-CEX affects students’ learning responses by triggering internal processes to do a follow-up on the feedback. External factors as a result of the mini-CEX function as summative assessment also affects the follow-up on feedback. Follow-up actions will result in various learning effects on the students. It is suggested that supervisors of clinical education are prepared to comprehend every factor influencing feedback on mini CEX to improve the students’ learning response.

### Conflict of Interest

The authors declare that they have no conflict of interest.
